# Neutrophil-to-Lymphocyte and Platelet-to-Lymphocyte Ratios in Nondialysis Chronic Kidney Patients

**DOI:** 10.1155/2021/6678960

**Published:** 2021-06-23

**Authors:** Gysllene M. C. Brito, Andrea M. M. Fontenele, Erika Cristina R. L. Carneiro, Iara Antonia L. Nogueira, Tamires B. Cavalcante, André A. M. Vale, Sally Cristina M. Monteiro, Natalino Salgado Filho

**Affiliations:** ^1^University Hospital of the Federal University of Maranhão, São Luís 65020-070, Brazil; ^2^Federal University of Maranhão, CEP: 65080-805, São Luís, Brazil

## Abstract

**Background:**

The Neutrophil-to-Lymphocyte Ratio (NLR) and the Platelet-to-Lymphocyte Ratio (PLR) are inflammatory biomarkers for several diseases, such as cancer and cardiovascular morbidities; however, there are currently few studies on kidney diseases. We aimed to evaluate nondialysis patients and determine the association of NLR and PLR with inflammation in these patients.

**Methods:**

A prospective cross-sectional study was conducted with 85 patients at different stages of chronic kidney disease (CKD), treated at the Kidney Disease Prevention Center of the University Hospital of the Federal University of Maranhão. This study included adult nondialysis patients diagnosed with CKD. The participants' blood samples were collected for a high-sensitivity C-reactive protein (hs-CRP) test and blood count. They were divided into two groups according to the presence or absence of inflammation based on the hs-CRP value (<0.5 mg/dL). NLR and PLR were calculated based on the absolute number of neutrophils, lymphocytes, and platelets and were compared between them and with hs-CRP. Statistical analysis was performed using the Stata software, with the Shapiro–Wilk, Mann–Whitney, Spearman's Correlation, and receiver operating characteristic curve tests. This study was approved by the local ethics committee.

**Results:**

The participants were categorized into two groups: with inflammation (*n* = 64) and without inflammation (*n* = 21). The mean age was 61.43 ± 14.63 y. The NLR and PLR values were significantly different between the groups with and without inflammation (*p*=0.045and *p*=0.004, respectively). However, only PLR showed a significant positive correlation with hs-CRP (*p*=0.015). The best cutoff point for NLR to detect inflammation was 1.98, with 76.19% sensitivity and 48.44% specificity. For PLR, it was 116.07, with 85.71% sensitivity and 51.56% specificity. There was no significant difference between the area under the NLR and PLR curve (0.71 vs. 0.64; *p*=0.186) for this population.

**Conclusions:**

This study showed that PLR was positively correlated with hs-CRP in nondialysis CKD patients and can be used to identify inflammation in this population.

## 1. Introduction

Chronic kidney disease (CKD) is characterized by progressive and irreversible loss of renal function. It is a major health issue worldwide, which leads to end-stage renal failure (ESRD) [1]. A systematic review and meta-analysis of observational studies revealed that CKD has an estimated global prevalence between 11 and 13%, and in Brazil, this prevalence, by population criteria, is between 3 and 6 millions of people [2, 3].

CKD has high morbidity and is associated with increased cardiovascular mortality, with 5 to 10 million annual deaths worldwide, followed by infections [1, 3, 4]. Inflammatory processes play a key role in chronic kidney disease and are considered a well-established risk factor for this pathology [5–8].

The chronic systemic inflammation in the CKD, sometimes referred to as low-grade chronic inflammation, is characterized by 2-3-fold increase of acute-phase protein and inflammatory mediators, slow developing, persistent, and of multifactorial origin [6, 8].

While the source(s) of chronic inflammation in CKD can vary, the negative implications of elevated inflammatory markers are clear, such as reduced renal function and high chances of mortality. Within the predialysis CKD population, the prevalence of inflammation is great and is an important indicator of patient health and outcome [5, 6].

It is predicted that early and specific detection of inflammation might improve the quality of life of those and decrease the rate of mortality and morbidity [4]. Nowadays, we have widely recognised diagnostic and monitoring markers, such as C-reactive protein (CPR), interleukins 1 and 6, and tumor necrosis factor *α* [9, 10].

However, in the present socioeconomic status, it is important that we seek cost-effective biological markers. Previous studies have demonstrated that the neutrophil-to-lymphocyte (NLR) ratio and platelet-to-lymphocyte ratio (PLR) have begun to be used with an indicator of systemic inflammation and have been widely studied in malignancies [11–15], hypertension, heart diseases, and vascular diseases [16, 17].

NLR and PLR are inexpensive, convenient, and measured easily and have demonstrated utility in stratifying mortality from cardiac events [14] and prognostic factor for cancer [15, 18–20], and it is reported that the NLR predicts the progression rate of stage 4 chronic kidney disease to dialysis [21]. Studies suggested that PLR was linked to inflammation and could predict mortality among hemodialysis (HD) patients [22, 23]. Their application for evaluating inflammation in dialysis patients has been addressed. However, the value of NLR and PLR in nondialysis patients remains unclear.

Therefore, the present study was designed to evaluate nondialysis patients and sought to determine the relationship of NLR and PLR with inflammation in these patients.

## 2. Materials and Methods

This was a cross-sectional study conducted with nondialysis chronic kidney disease (CKD) patients treated at the Kidney Disease Prevention Center (CPDR) at the University Hospital of the Federal University of Maranhão (HUUFMA).

A nonprobabilistic sample was formed by male and female adult and elderly patients with a diagnosis of CKD who were not on dialysis and were treated at the CPDR outpatient clinic during a 12-month period (September 2016 to August 2017). The exclusion criteria were pregnant women, patients with amputations, with only one kidney, with a history of hospitalization and surgeries (including oral cavity) in the 3 months prior to the beginning of data collection, and history of dialysis, liver failure, chronic consuming diseases (e.g., cancer, severe heart failure, and acquired immunodeficiency syndrome), and/or infectious diseases.

Participants, regardless of skin color or financial status, were selected by a doctor during outpatient care and referred to pharmaceutical assistance. During the pharmaceutical appointment, patients were informed about the study and invited to participate. A total of 101 patients of both sexes with a diagnosis of nondialysis CKD agreed to participate in this study [24]. However, due to insufficient data and/or patient dropout, the final sample number was 85.

This study was conducted in two stages. The first stage was an interview to collect demographic, socioeconomic, lifestyle, and comorbidities data. In the second stage, data were collected from electronic medical records and blood samples to perform the hs-CRP and complete blood count test.

The hs-CRP test was performed using serum with the immunoturbidimetry methodology with a Roche Cobas 6000 analyzer (Roche Holding AG, Basel, Switzerland). The complete blood count was performed using the Advia 2120 Hematology System (Siemens Healthineers, Erlangen, Germany).

The levels of urea and creatinine were obtained from the HUUFMA electronic records. The glomerular filtration rate value was calculated using the Chronic Kidney Disease Epidemiology Collaboration Creatinine Equation (2009) through the mobile application eGFR (version 2.3; Fresh Mint Labs, NY, USA), based on data from the National Kidney Foundation. NLR and PLR were obtained by dividing the absolute neutrophil count by lymphocytes and between platelets and lymphocytes, respectively.

Participants were categorized according to the presence of low-grade inflammation based on the hs-CRP value, a gold-standard method for the detection of inflammation. As the cutoff point for hs-CRP, participants with no inflammation were those who obtained a result <0.5 mg/dL [25].

The comorbidities found were arterial hypertension, diabetes mellitus, and cardiovascular disease and were determined by previous self-reported diagnosis.

Data were tabulated in Microsoft Excel (Microsoft Corporation, Albuquerque, NM, USA), and the statistical analysis was performed using Stata software (version 14; StataCorp, College Station, TX, USA). Initially, descriptive analyses were performed using absolute and relative frequencies for categorical and mean variables, standard deviation for numerical variables with normal and median distribution, and interquartile range for those without normal distribution. Normality was assessed using the Shapiro–Wilk test. For inferential analysis, the chi-square and Fisher's exact tests were used to compare categorical variables between groups with and without inflammation, the Mann–Whitney *U* test for the evaluation of NLR and PLR as inflammatory markers in people with CKD, and the coefficient of Spearman's correlation to compare the numerical distributions of each marker with the hs-CRP. The Youden test was used in the receiver operating characteristic (ROC) curve to identify the cutoff points of the markers with the best sensitivity and specificity. The area under the curve (AUC) was used to assess the markers' performance and comparison. The level of significance used for the statistical tests was 5%. The data are presented in tables and graphs.

This study was approved by the local ethics committee under number 2.015.866, with all participants signing an informed consent form.

## 3. Results

The final sample consisted of 85 participants categorized into two groups according to the presence of inflammation (24.7%) and absence of inflammation (75.3%). The mean age was 61.43 ± 14.63 y, with a predominance of female patients (55.29%). The most prevalent comorbidity found was arterial hypertension (92.94%) (Table 1).

**Figure 1:**
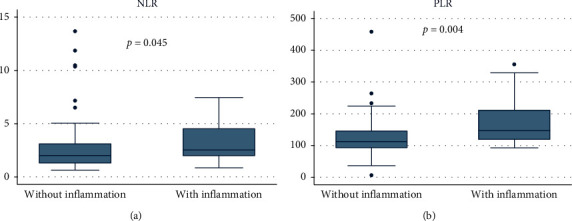
Comparison between groups with and without inflammation based on NLR and PLR in nondialysis patients with chronic kidney disease, using the Mann–Whitney *U* test; NLR: neutrophil-to-lymphocyte ratio; PLR: platelet-to-lymphocyte ratio.

The hs-CRP value (median of 0.14 mg/dL between the participants) was used as a cutoff point to separate the group with and without inflammation, as described in the methodology. The median values of the group without inflammation were 0.09 mg/dL and 0.88 mg/dL for the group with inflammation (*p* < 0.001). The levels of urea and creatinine and the glomerular filtration rate did not show significant differences between the groups. NLR and PLR showed median levels of 2.11 (1.41–3.36) and 121.95 (94.72–157.45), respectively, with a statistically significant difference between groups for NLR (*p*=0.045) and PLR (*p*=0.004) (Figure 1).  Table 2 shows the results of the laboratory tests and their statistical analysis.

The results showed a statistically significant difference between the groups with and without inflammation based on NLR and PLR values (*p*=0.045 and *p*=0.004, respectively), confirming that both are markers that alter or stand out in inflammatory processes for nondialysis patients with CKD, as shown in Figure 1.

**Figure 2:**
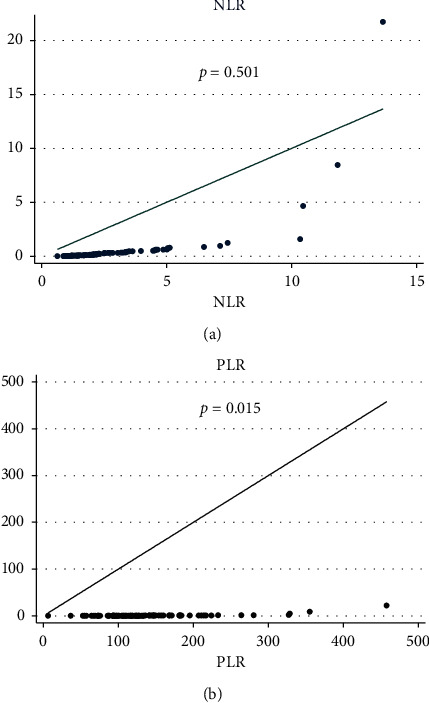
Correlation between NLR, PLR, and hs-CRP in nondialysis patients with chronic kidney disease using Spearman's correlation test. NLR: neutrophil-to-lymphocyte ratio; PLR: platelet-to-lymphocyte ratio; and hs-CPR: high sensitivity C-reactive protein.

A statistically significant positive correlation was found between PLR and hs-CRP (*p*=0.01; *r* = 0.261). However, this correlation did not occur for NLR and hs-CRP (*p*=0.501; *r* = 0.074), as shown in Figure 2.

**Figure 3:**
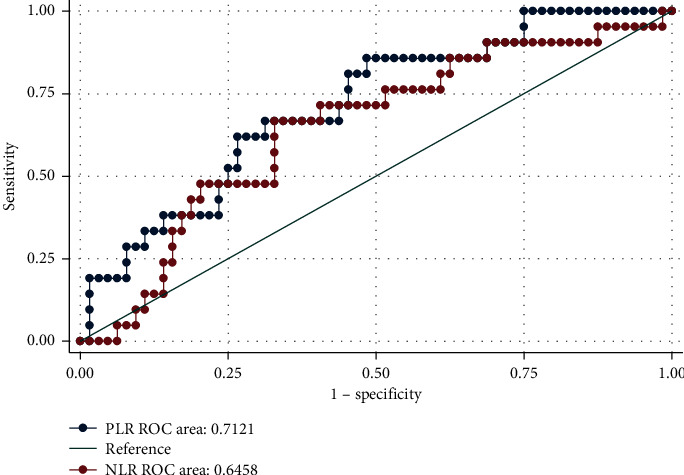
Analysis of the ROC curve for different cutoff points for NLR and PLR in patients with nondialysis chronic kidney disease using the ROC curve and area under curve (AUC) test. ROC: receiver operating characteristic; NLR: neutrophil-to-lymphocyte ratio; and PLR: platelet-to-lymphocyte ratio.

The analysis of the ROC curve showed that the best cutoff point for NLR, with 76.19% sensitivity and 48.44% specificity, was 1.98. The best cutoff point for PLR, with 85.71% sensitivity and 51.56% specificity, was 116.07. The difference in the AUC between NRL and PLR was not statistically significant (AUC: 0.64 vs. AUC: 0.71, respectively; *p*=0.186) (Figure 3).

## 4. Discussion

NLR is a parameter that provides information on both inflammation and the stress response. As for PLR, a high platelet count and a low level of lymphocytes are associated with different cardiovascular outcomes [26]. In this context, studies show that high levels of NLR and PLR are associated with clinical pathological conditions in certain neoplasms [27–29] and in cardiovascular diseases [30–32]. However, there are few studies to date on these markers for CKD.

The objective of this study was to evaluate nondialysis CKD patients and determine the association of NLR and PLR with inflammation in these patients, and the results showed that both biomarker values significantly increased in patients with inflammation.

The study by Chávez Valencia [33] pointed out that these markers can be used to identify hemodialysis patients with inflammation. Ahbap et al. [22], who worked with 100 patients in stage 5 CKD on maintenance hemodialysis, concluded that both NLR and PLR had higher levels in patients with inflammation. Both studies highlighted the advantage of these markers as they are simple and low-cost methods.

Some studies that focused only on NLR outlined its advantages compared to other inflammatory markers. Malhotra et al. [34] concluded that NLR could be a potential substitute marker for hs-CPR in hemodialysis patients, since it is a useful systemic inflammation test, especially in places with limited resources. Okyay et al. [35] stated that the determination of NLR values is easy and inexpensive and can provide significant information about the inflammatory state in CKD.

In some studies, NRL stood out as an inflammatory marker, reaching higher levels than other markers such as hs-CPR and interleukins and in some outcomes as an indicator of acute kidney injury in patients with sepsis and as an indicator of cardiovascular events in patients with end-stage renal disease [36, 37]. Yoshtomi et al. [38] showed that higher NLR was associated with worse renal outcomes, indicating that it is useful as a prognostic marker. Yuan et al. [39] suggested that NLR could be used in risk assessment for stage 4 patients to progress to renal replacement therapy. Considering the aforementioned facts, NLR can be considered a promising inflammatory biomarker in renal disease patients.

The data presented here corroborate the findings of these authors, showing that NLR changes in the group with nondialysis CKD, but in the studied population, it was not positively correlated with hs-CPR.

On the other hand, few studies have shown evidence of the use of PLR as an inflammatory marker in this population. It is considered a recently defined hematological parameter, which is associated with both aggregation and inflammation pathways and may be more valuable than the isolated platelet or lymphocyte count [40].

There was a positive correlation between PLR and hs-CPR, a gold-standard method for the detection of inflammation in this population. Similar findings were presented in a study conducted in Turkey, where patients on hemodialysis and peritoneal dialysis had higher PLR than NLR, in addition to being positively correlated with other inflammatory markers, such as cytokines and tumor necrosis factor-alpha [41], highlighting another potential inflammatory biomarker that can be easily used in clinical practice.

Chávez Valencia et al. [33] concluded that PLR is correlated with some inflammatory parameters (CRP and interleukin 6) and was better than NLR in this sense. Okyay et al. [35] found that, in CKD patients, both NLR and PLR correlated positively with other inflammatory markers, such as hs-CPR.

In this context, NLR and PLR show a great advantage compared to other markers in the evaluation of inflammation since it is a simple, relatively inexpensive, and universally available method [22] that could be used by healthcare professionals as a first method of assessing inflammation before other more expensive and invasive procedures [41].

As for the determination of the markers' cutoff points, the best sensitivity and specificity values were 1.98 for NLR and 116.07 for PLR. The literature presents great variability as for cutoff values for these markers. Tonyali et al. [42] found a cutoff point of 3.18 for NLR in patients in the postoperative period of partial or radical nephrectomy, and for Yilmaz et al. [37], who worked with cases of severe sepsis and acute kidney injury, the cutoff point for NLR was 10.5.

As for PLR, a cutoff value of 136.85 was found in patients who underwent cardiovascular surgery [40]. The study by Cetinkaya et al. [43] with patients who underwent nephrolithotomy determined the cutoff value of 114.1 and added that patients with PLR above that value are more likely to develop systemic inflammatory response syndrome.

The difference of performance and comparison between the markers was not significant in this study. Li [44], who also analyzed nondialysis renal disease patients, found the ideal cutoff points for NLR and PLR to be 5.07 and 163.80, respectively. They showed that NLR and PLR also positively correlated with hs-CPR but were not significant enough to replace it. In this study, however, only PLR was positively correlated with hs-CPR, showing that it can be an additional method of assessing inflammation in this population, but not as a substitute method.

These findings indicate that studies with larger, randomized, and multicenter samples are still needed to better assess the performance of these markers in nondialysis renal disease patients.

The present study has as limitations the fact that it is cross sectional with local sampling that was nonprobabilistic and relatively small for some statistical analyses, and a cause-and-effect relationship cannot be defined in the findings of this study. On the other hand, this study was conducted in a reference center for the prevention and treatment of CKD in a capital city that concentrates a representative sample of the population of the state. In summary, the data from the present study were able to confirm the association between the values of NLR and PLR and the presence of inflammation in this population. Nevertheless, the literature has few studies on this theme, showing the need for larger, controlled studies to analyze the use of these markers in patients with CKD and their implementation in clinical practice.

## 5. Conclusions

The study showed that PLR was positively correlated with hs-CPR in nondialysis CKD patients and, thus, can be used to identify inflammation in this population.

## Figures and Tables

**Table 1:** Demographic and clinical characteristics of patients at the Kidney Disease Prevention Center of the University Hospital of the Federal University of Maranhão, according to hs-CRP groups in nondialysis patients, São Luís, MA, Brazil, 2019.

Variables	Total, *n* = 85		Groups			
			Without inflammation (hs-CPR<0.5 mg/dL), *n* = 64		With inflammation (hs-CPR >0.5 mg/dL), *n* = 21	
	*N*	%	*N*	%	*N*	%	*p* value
*Sex*							
Male	38	44.71	26	40.63	12	57.14	0.186
Female	47	55.29	38	59.38	9	42.86	

*Smoking*							
Yes	2	2.35	2	3.13	0	0	0.468
No	44	51.76	35	54.69	9	42.86	
Stopped	39	45.88	27	42.19	12	57.14	

*SAH*							
Present	79	92.94	61	95.31	18	85.71	0.157
Absent	6	7.06	3	4.69	3	14.29	

*DM*							
Present	40	47.06	28	43.75	12	57.14	0.286
Absent	45	52.94	36	56.25	9	42.86	

*CVD*							
Present	22	25.88	16	25	6	28.57	0.746
Absent	63	74.12	48	75	15	71.43	

SAH = systemic arterial hypertension, DM = diabetes mellitus, CVD = cardiovascular disease, hs-CRP = high-sensitivity C-reactive protein.

**Table 2:** Characteristics and laboratory findings according to hs-CRP groups in nondialysis patients at the Kidney Disease Prevention Center of the University Hospital of the Federal University of Maranhão, São Luís, MA, Brazil, 2019.

Variables	Total, *n* = 85	Groups	
		Without inflammation (hs-CPR <0.5 mg/dL), *n* = 64	With inflammation (hs-CPR >0.5 mg/dL), *n* = 21
	Median (IQ_25–75%_)	Median (IQ_25–75%_)	Median (IQ_25–75%_)	*p* value
Urea (mg/dL)	45.00 (34.00–69.00)	44.50 (33.50–73.55)	46.00 (37.00–67.00)	0.995
Creatinine (mg/dL)	1.70 (1.47–1.97)	1.70 (1.46–1.97)	1.69 (1.52–1.95)	0.862
GFR (mL/min)	42.60 (34.50–49.60)	42.60 (34.85–50.15)	43.80 (34.00–49.00)	0.906
hs-CPR (mg/dL)	0.14 (0.05–0.47)	0.09 (0.04–0.21)	0.88 (0.63–1.71)	0.001
NLR	2.11 (1.41–3.36)	2.03 (1.31–3.12)	2.52 (1.98–4.52)	0.045
PLR	121.95 (94.72–157.45)	112.75 (93.00–145.52)	146.43 (119.11–210.92)	0.003

GFR = glomerular filtration rate, hs-CPR: high sensitivity C-reactive protein, PLR = platelet-to-lymphocyte ratio, NLR = neutrophil-to-lymphocyte ratio.

## Data Availability

The data used to support the findings of this study are available from the corresponding author upon request.
